# Variable selection from a feature representing protein sequences: a case of classification on bacterial type IV secreted effectors

**DOI:** 10.1186/s12859-020-03826-6

**Published:** 2020-10-27

**Authors:** Jian Zhang, Lixin Lv, Donglei Lu, Denan Kong, Mohammed Abdoh Ali Al-Alashaari, Xudong Zhao

**Affiliations:** 1 College of Artificial Intelligence, Wuxi Vocational College of Science and Technology, No. 8 Xinxi Road, Wuxi, 214028 China; 2 College of Information and Computer Engineering, Northeast Forestry University, No. 26 Hexing Road, Harbin, 150040 China

**Keywords:** Feature selection, Variable importance, Accumulated scoring, Classification, Bacterial type IV secreted effectors

## Abstract

**Background:**

Classification of certain proteins with specific functions is momentous for biological research. Encoding approaches of protein sequences for feature extraction play an important role in protein classification. Many computational methods (namely classifiers) are used for classification on protein sequences according to various encoding approaches. Commonly, protein sequences keep certain labels corresponding to different categories of biological functions (e.g., bacterial type IV secreted effectors or not), which makes protein prediction a fantasy. As to protein prediction, a kernel set of protein sequences keeping certain labels certified by biological experiments should be existent in advance. However, it has been hardly ever seen in prevailing researches. Therefore, unsupervised learning rather than supervised learning (e.g. classification) should be considered. As to protein classification, various classifiers may help to evaluate the effectiveness of different encoding approaches. Besides, variable selection from an encoded feature representing protein sequences is an important issue that also needs to be considered.

**Results:**

Focusing on the latter problem, we propose a new method for variable selection from an encoded feature representing protein sequences. Taking a benchmark dataset containing 1947 protein sequences as a case, experiments are made to identify bacterial type IV secreted effectors (T4SE) from protein sequences, which are composed of 399 T4SE and 1548 non-T4SE. Comparable and quantified results are obtained only using certain components of the encoded feature, i.e., position-specific scoring matix, and that indicates the effectiveness of our method.

**Conclusions:**

Certain variables other than an encoded feature they belong to do work for discrimination between different types of proteins. In addition, ensemble classifiers with an automatic assignment of different base classifiers do achieve a better classification result.

## Background

Feature extraction from protein sequences plays an important role in protein classification [1–4] of many areas, such as identification of plant pentatricopeptide repeat coding protein [5], prediction of bacterial type IV secreted effectors [6–9], identification of heat shock protein [10], prediction of mitochondrial proteins [11], etc. In general, prevailing encoding approaches of protein sequences for feature extraction include pseudo-amino acid composition (PseAAC) [10–22], position-specific scoring matrix (PSSM) [7, 23–32], position-specific iterated blast (PSI-BLAST) [33–37] etc.

However, several problems do still exist and are listed as follows. First of all, it needs to be decided which encoding approach is more effective. In fact, this problem can be solved according to the results of classification on each encoding approach using various computational methods (i.e., known as classifiers in the field of machine learning or pattern recognition). In other words, the encoding approach corresponding to the most accurate classification result should be considered. Prevailing classifiers including random forest or decision tree classifier (RF or DTC) [1, 38], gradient boosting machine (GBM) [39, 40], k-nearest-neighbor (kNN) [41, 42], linear discriminant analysis (LDA) [43, 44], logistic regression (LR) [45], multi-layer perceptron (MLP) [46, 47], naive bayesian (NB) [5, 48], support vector machine (SVM) [49, 50] are credible.

Secondly, it needs to be discussed whether protein classification is predictive or not, which is a little confused. Actually, classification labels have commonly been assigned to protein sequences in advance. If these labels are definitive, i.e., having been certified by biological experiments in advance, there won’t be any need to predict the category of a protein sequence again. Conversely, unsupervised learning (e.g. clustering) rather than supervised learning (e.g. classification) should be considered, since these labels are undetermined. And that corresponds to protein prediction. However, prevailing methods are always confusing protein classification an protein prediction.

Thirdly, the extracted feature using an encoding approach is considered to be entirely effective. In fact, there may be only parts of the extracted feature that are effective. However, this phenomenon has been subjectively neglected. As a result, it is an important issue how to select certain components or variables from a feature that really helps to recognize proteins with specific functions. In other words, variable selection from a feature representing protein sequences is a new problem probably not yet avoidable, which may be more helpful to classification of different protein sequences.

**Fig. 1:**
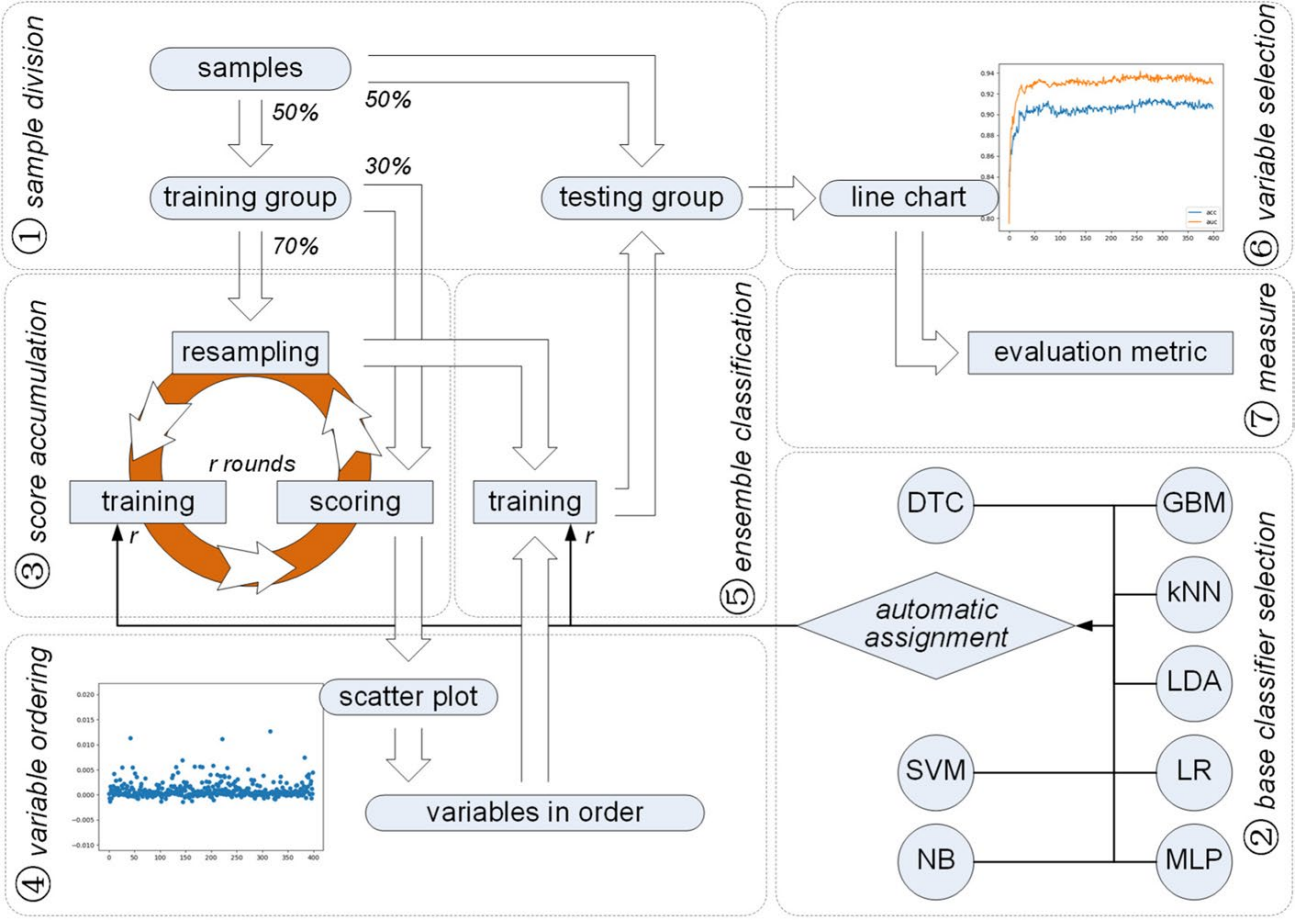
A framework of variable selection for identifying different proteins

In this paper, we propose a new method for variable selection from an encoded feature. The selection of a feature from an encoding approach is excluded from our method. Besides, no prediction work is executed. Focusing on components or variables of an encoded feature, we implement our method at seven steps as shown in Fig. 1. First of all, samples are divided in balance, which constitute a training and testing group. Secondly, a base classifier is automatically assigned in every resampling of the training group. Thirdly, the score of each variable in an encoded feature is accumulated through *r* rounds of resampling, training and scoring. Fourthly, a scatter plot and corresponding order of variables with their accumulated scores in a descending order are obtained. Fifthly, *r* rounds of training are made on resampling samples to achieve ensemble classifiers in each dimension (i.e., from one to full dimension of the encoded feature) according to variables incrementally added in the descending order. Sixthly, variable selection is accomplished using a line chart derived from the classification results on the testing group. Seventhly, evaluation metrics are made to estimate the effectiveness of selected variables. Experiments are made on the benchmark dataset [51] to identify bacterial type IV secreted effectors from protein sequences, which indicates the effectiveness of our method. More details can be seen in the following parts of this paper.

## Results

In this section, we take a benchmark dataset [51] as a case to evaluate the performance of our proposed method. The dataset is composed of 1947 protein sequences across multiple bacterial species, categorized into two groups, i.e., 399 type IV secreted effectors (T4SE) as the positive samples and 1548 non-T4SE as the negative samples. The 1947 protein sequences are randomly divided into two subsets for training and testing, respectively. The training set consists of 973 sequences, among which 199 T4SE and 774 non-T4SE sequences are randomly selected from positive and negative samples, respectively. The left 200 T4SE and 774 non-T4SE samples constitute the testing set. Besides, we choose PSSM, which is composed of 400 variables, as the encoded feature. Following the procedure shown in Fig. 1, the experimental results of score accumulation, ensemble classification, variable selection and the corresponding classification results are listed as follows.

### Results of score accumulation

**Fig. 2:**
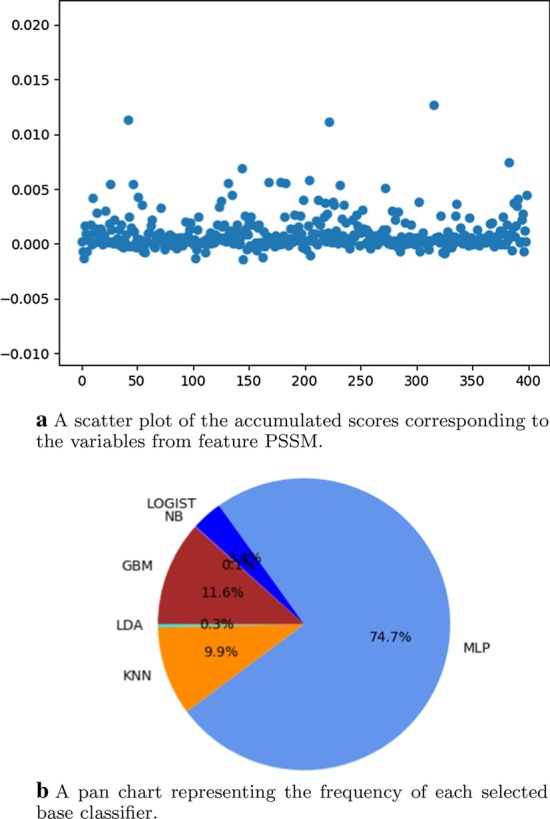
Results of score accumulation

We randomly extract 70% of samples from the training set and choose a classifier with the lowest classification error rate as the base classifier at each round. Meanwhile, scores representing the importance of variables are calculated. After 1000 rounds of resampling, training and scoring (i.e., $r=1000$), we obtain the accumulated scores of each variable from PSSM. The corresponding scatter plot is shown in Fig. 2a. Its horizontal and vertical coordinates correspond to 400 variables and their accumulated scores, respectively. In addition, the frequency of each selected base classifier is illustrated in Fig. 2b.

**Fig. 3:**
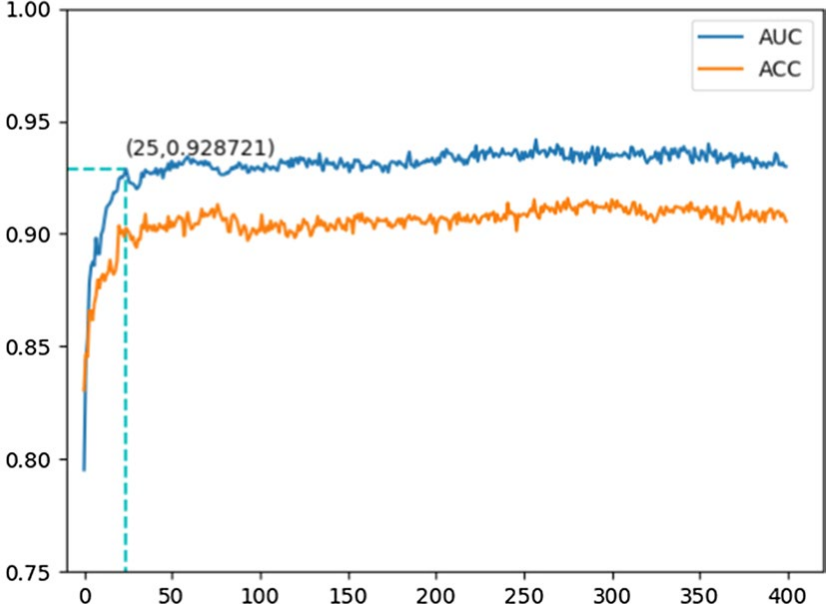
A line chart of ACCs and AUCs corresponding to the incrementally added variables from feature PSSM with their accumulated scores in a descending order

It can be seen in Fig. 2a that the accumulated scores are all relatively low. Since the accumulated scores of the 400 variables have no apparent distinction, all these variables are considered to be enumerated at the following step instead of selecting variables with high accumulated scores, as having been stated in [52].

In Fig. 2b, it can be seen that MLP is automatically assigned as the base classifier for 74.7% of 1000 round resampling. On the contrary, DTC and SVM have never been selected for score accumulation.

### Results of ensemble classification on testing group

The ensemble classifiers have been built using 1000 rounds of resampling and training on the training set in each dimension, with 400 variables incrementally added in the descending order according to their accumulated scores. Then, the 400 ensemble classifiers, each of which keeps 1000 base classifiers, are applied to the testing set. As a result, a line chart (see Fig. 3) is obtained with its horizontal corresponding to the dimensions with variables incrementally added in the descending order according to their accumulated scores. The vertical coordinates are referred to the Acc and AUC values in the incremental dimensions. It can be seen in Fig. 3 that it is the first 25 variables with descending accumulated scores that form a 25-D feature from PSSM for effective identification of T4SE.

**Table 1:** Quantitative results by incrementally adding the variables after the 25th one of 400 variables in a descending order according to their accumulated scores and the comparison results shown in reference [6]

Measure	Max value	Mean value	Min value	Results in [6]		NB	kNN	LR	RF	ERT	SVM	XGB	GBM
Acc	0.910	0.906	0.893	Training set	1st stage	0.732	0.855	0.879	0.885	0.894	0.902	0.901	0.905
					2nd stage	0.909	0.910	0.911	0.904	0.889	0.906	0.907	0.906
				Testing set	2nd stage	0.938	0.935	0.944	0.938	0.940	0.945	0.924	0.931
AUC	0.940	0.932	0.918	Training set	1st stage	0.812	0.907	0.906	0.921	0.925	0.935	0.927	0.929
					2nd stage	0.927	0.925	0.929	0.906	0.812	0.935	0.921	0.907

Moreover, some quantitative results are shown to indicate the effectiveness of the obtained 25 variables for classification. Table 1 lists the max, mean and min values of Accs and AUCs by incrementally adding the variables after the 25th one of the 400 variables in a descending order according to their accumulated scores. It can be indicated that features enlarged with higher dimensions can achieve only similar Accs and AUCs as the 25 variables do.

**Table 2:** Quantitative results among ensemble classification with automatic assignment of base classifiers and the ensemble classifier with single base classifiers

Classifier	Dimension	Confusion matrix			Positive class	Precision	Recall	F1-measure
Automatic assignment	400	Classified as	Non-T4SE	T4SE	Non-T4SE	0.943	0.938	0.940
		Label non-T4SE	726	48	T4SE	0.765	0.780	0.772
		Label T4SE	44	156	Weighted average	0.906	0.906	0.906
Automatic assignment	25	Classified as	Non-T4SE	T4SE	Non-T4SE	0.944	0.933	0.938
		Label non-T4SE	722	52	T4SE	0.751	0.785	0.768
		Label T4SE	43	157	Weighted average	0.904	0.903	0.903
DTC	25	Classified as	Non-T4SE	T4SE	Non-T4SE	0.925	0.950	0.937
		Label non-T4SE	735	39	T4SE	0.782	0.700	0.739
		Label T4SE	60	140	Weighted average	0.896	0.899	0.896
GBM	25	Classified as	Non-T4SE	T4SE	Non-T4SE	0.926	0.953	0.939
		Label non-T4SE	738	36	T4SE	0.797	0.705	0.748
		Label T4SE	59	141	Weighted average	0.900	0.902	0.900
kNN	25	Classified as	Non-T4SE	T4SE	Non-T4SE	0.931	0.919	0.925
		Label non-T4SE	711	63	T4SE	0.700	0.735	0.717
		Label T4SE	53	147	Weighted average	0.884	0.881	0.882
LDA	25	Classified as	Non-T4SE	T4SE	Non-T4SE	0.927	0.925	0.926
		Label non-T4SE	716	58	T4SE	0.713	0.720	0.716
		Label T4SE	56	144	Weighted average	0.883	0.883	0.883
LR	25	Classified as	Non-T4SE	T4SE	Non-T4SE	0.883	0.957	0.919
		Label non-T4SE	741	33	T4SE	0.756	0.510	0.609
		Label T4SE	98	102	Weighted average	0.857	0.865	0.855
MLP	25	Classified as	Non-T4SE	T4SE	Non-T4SE	0.930	0.946	0.938
		Label non-T4SE	732	42	T4SE	0.775	0.725	0.749
		Label T4SE	55	145	Weighted average	0.898	0.901	0.899
NB	25	Classified as	Non-T4SE	T4SE	Non-T4SE	0.942	0.875	0.907
		Label non-T4SE	677	97	T4SE	0.620	0.790	0.695
		Label T4SE	42	158	Weighted average	0.876	0.858	0.863
SVM	25	Classified as	Non-T4SE	T4SE	Non-T4SE	0.925	0.924	0.924
		Label non-T4SE	715	59	T4SE	0.706	0.710	0.708
		Label T4SE	58	142	Weighted average	0.880	0.880	0.880

### Classification results of the selected variables

In order to show the effectiveness of the selected 25 variables, the confusion matrix, *Precision*, *Recall* and $F1-measure$ are calculated in order to make a quantitative comparison. In addition, ROCs together with AUCs are listed as qualitative results.

**Fig. 4:**
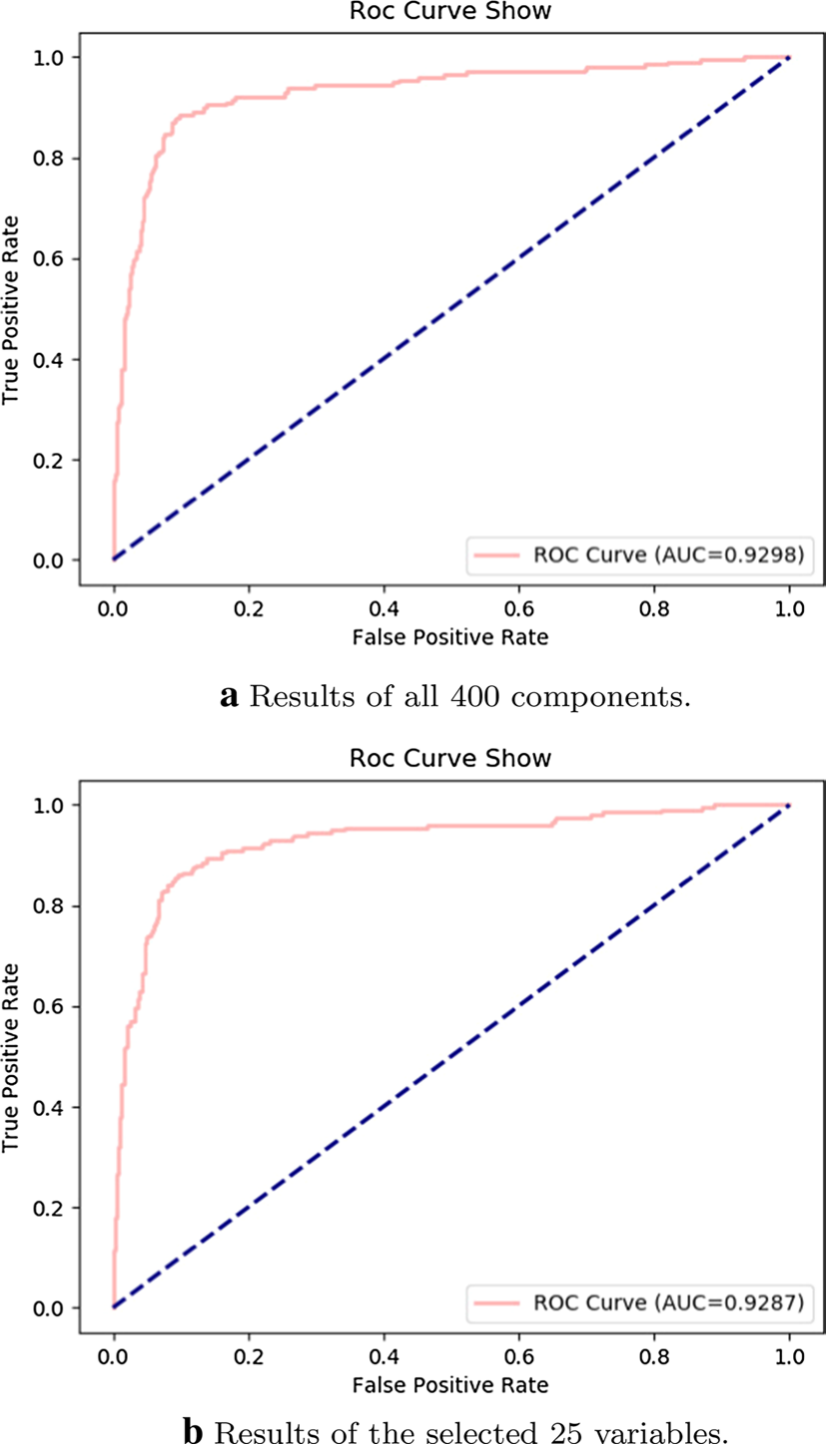
Comparison results of ROC and AUC between all 400 components and the selected 25 variables using ensemble classification

Results of ROC and AUC between the first 25 selected variables and all 400 components of PSSM using ensemble classification are shown in Fig. 4. The similar ROC curves and AUC values indicate that the selected 25 variables keep a comparable classification capability with PSSM.

**Fig. 5:**
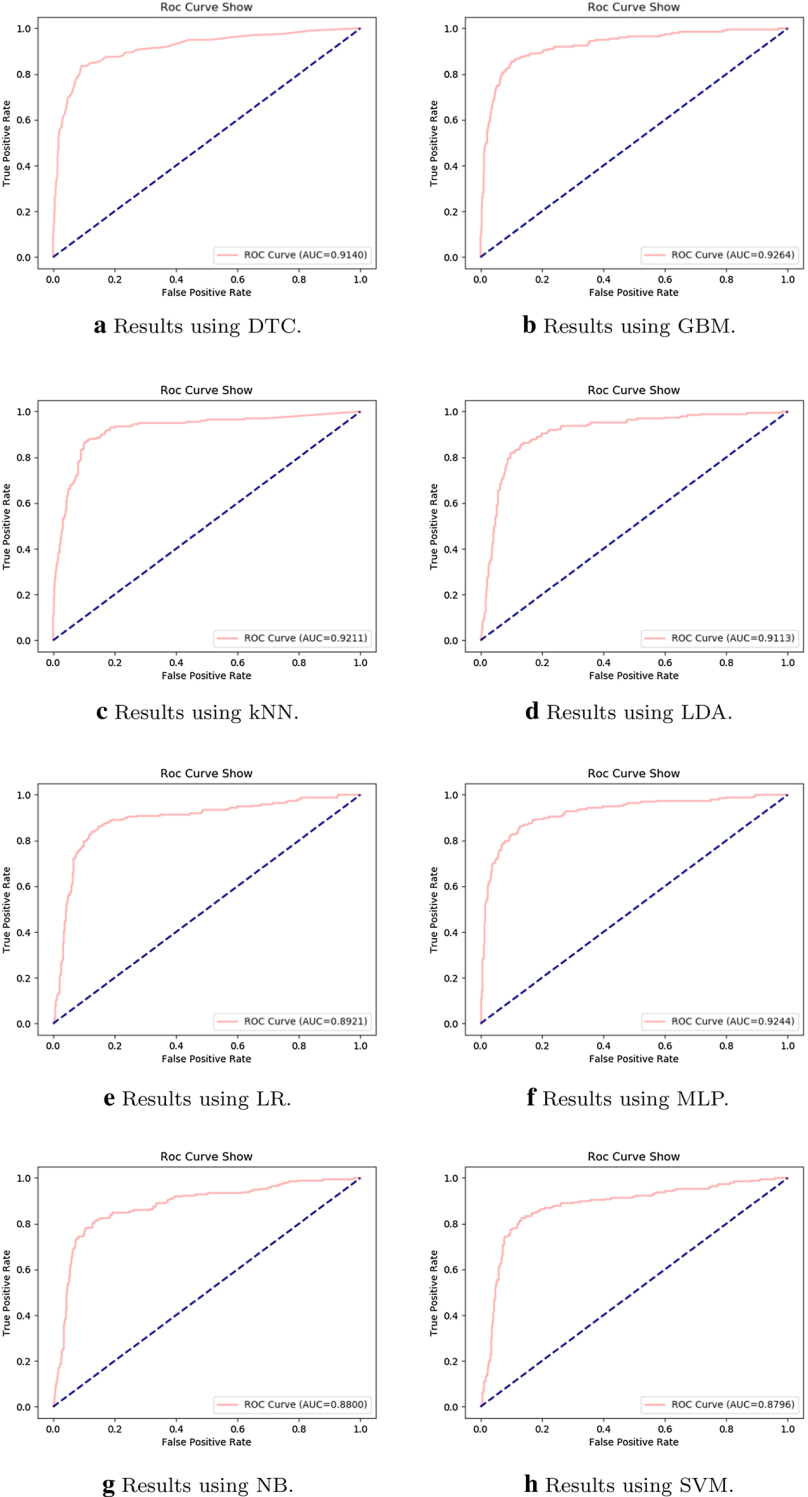
Results of ROC and AUC using different base classifiers with the selected 25 variables

Besides, results of ROC and AUC using the ensemble classifier consisting of 1000 single base classifiers with the selected 25 variables are illustrated in Fig. 5. By making a careful comparison between Figs. 4b and 5, it can be seen that ensemble classification with automatic assignment of base classifier keeps a better ROC curve and AUC value (i.e., 0.9287).

Moreover, quantitative results among ensemble classification with automatic assignment of a base classifier and the ensemble classifier with a single base classifier are listed in Table 2. It can be seen that the ensemble classifier with automatic assignment of a base classifier on the 25 selected variables keeps a high TP (i.e., 157) compared with most of the other classification strategies. Besides, it has better values of *Precision*, *Recall* and $F1-measure$ (i.e., 0.904, 0.903 and 0.903) compared with the other ensemble classifier with a single base classifier on the 25 selected variables. As to the results of the ensemble classifier with automatic assignment of a base classifier on PSSM (i.e., 400 components), the selected 25 variables can also achieve comparable results.

## Discussions

Experimental results have indicated the effectiveness of variable selection from the encoded feature PSSM. In this section, we will further discuss the special composition of our variable selection method and the classification results.

### Purpose of using base classifier selection

The automatic assignment of a base classifier is creative in this paper. Giving consideration to the sample distribution of resampling, we designed the strategy of automatic assignment. Due to the limited sample size, resampling is only an approximation to the population. In our previous work, it has been pointed out that different base classifiers should be considered according to various sample distributions [52]. However, the base classifier was interactively appointed in [52]. The pan chart in Fig. 2b can also show this phenomenon, which indicates that base classifiers selected at a higher percentage may fit the population better. Besides, quantitative results listed in Table 2 indicate the power of using automatic assignment compared with the interactive appointment of a base classifier.

### Purpose of using a line chart for variable selection

In fact, a line chart shown in Fig. 3 is a semi-automatic way for variable selection. Here, it goes against the interactive way that uses a manual selection within a table or on a 2-D scatter plot [52]. Also, it abandons the automatic way of automatic clustering [53] on accumulated scores. Due to the limited distribution of the accumulated scores with relatively low values, the variables have no apparent distinction. It probably means variables are highly correlated. In that case, variable orders instead of values are to be considered.

### Comparisons between classification results

We compared the classification results of our method with PredT4SE-Stack [6]. As shown in Table 1, the max, mean and min values of Accs and AUCs on the testing set exhibit a better result than the classification results on its training set using PreT4SE-Stack. The best classification Acc value using PredT4SE-Stack on its training set is 0.911. Most of the other meta-classifier got a lower Acc value and AUC value than the mean Acc and AUC obtained using our method. However, classification results of Pred4SE-Stack on its testing set are better than those using our method. In fact, the classification results on its testing set are even better than those on its training set. By careful observation, it is found that parameters of base classifiers are manually set every time in [6]. That’s why PredT4SE-Stack gets better classification results on its testing set. Anyway, seeking better classification results by setting parameter values doesn’t make any sense for variable selection.

## Conclusion

In order to solve the problem of variable selection from an encoded feature representing protein sequences, we propose a variable selection method based on ensemble classification with an automatic assignment of base classifiers. Variable ordering is obtained according to score accumulation on training samples. Then, ensemble classifiers are established from one to all dimensions of the encoded feature according to variables incrementally added in the descending order. Using the ensemble classifiers on testing samples, a line chart is drawn for variable selection. Ultimately, evaluation metrics are made to estimate the effectiveness of selected variables. Taking a dataset containing protein sequences categorized into T4SE and non-T4SE group as a case, the performance of the proposed method is evaluated.

## Methods

In this section, we will expound our method in detail. As illustrated in Fig. 1, our method has seven steps, each of which is framed in a dashed box. Each step which keeps its name labeled within the dashed box, corresponds to a following subsection.

### Sample division

We make a balanced sample division at the first step. That is, samples derived from a category are divided into two equivalent parts. As a result, half of the samples from different categories form a training group; while, the left ones constitute a testing group. It is noteworthy that this sample division should be performed in a completely random way.

### Base classifier selection

A base classifier is automatically assigned at the second step. We make a set of base classifiers including decision tree classifier (DTC), gradient boosting machine (GBM), k-nearest-neighbor (kNN), linear discriminant analysis (LDA), logistic regression (LR), multi-layer perceptron (MLP), naive bayesian (NB) and support vector machine (SVM). Each one is equally assigned in an automatic way corresponding to every round of resampling and training module. In each round *j*, 70% of training samples are randomly selected in a full dimension of an encoded feature for training each base classifier. The remaining 30% of training samples are regarded as the out-of-bag samples for calculation of the classification error rate $Err_{j}$, as is expressed in Eq. (1). The base classifier with the lowest classification error rate is automatically assigned in round *j*.

### Score accumulation

Score accumulation is made at the third step. Once a base classifier is automatically assigned according to the classification error rate calculated on the out-of-bag samples in round *j*, permutations are to be made. As to each variable *i* of the encoded feature, only one-time permutation of the expression values from the out-of-bag samples is performed. The corresponding classification error rate is denoted as $Err^{0}_{j}(i)$. Accordingly, a score representing the importance of variable *i* is expressed as $score_{j}(i) = Err^{0}_{j}(i)-Err_{j}$. After *r* rounds of resampling, training and scoring, the accumulated score of variable *i* is expressed as $\sum _{j=1}^{r}score_{j}(i)/r$.

### Variable ordering

Variables are reordered at the fourth step. A 2-D scatter plot is to be made with its horizontal and vertical coordinates corresponding to the variable indices and the accumulated scores, respectively. Besides, variables are to be sorted in a descending order according to the accumulated scores. If the accumulated scores of the variables have no distinction (i.e., the accumulated scores are all relatively low), all the variables rather than the significant variables selected using previously proposed clustering method [53] are to be enumerated at the following step.

### Ensemble classification

Ensemble classifiers are established at the fifth step. Again, *r* rounds of resampling and training are performed to achieve ensemble classifiers in each dimension according to variables incrementally added in their descending order. As to 1-D space, the variable with the highest accumulated score is considered. At each round of resampling, the base classifier with the lowest classification error rate is trained. Altogether, *r* base classifiers are selected as the ensemble classifier in 1-D space. This procedure is repeated with a variable keeping a lower accumulated score added, until the full dimension of the encoded feature or the full dimension of significant variables has been considered.

### Variable selection

Variable selection is accomplished at the sixth step. In each dimension, the established ensemble classifier is applied to the testing samples. The accuracy (Acc) expressed in Eq. (2) and the area under curve (AUC) of the receiver operating characteristic (ROC) are calculated. Accordingly, a line chart is obtained with its horizontal and vertical coordinates corresponding to the variable indices in their descending order and the corresponding Accs and AUCs in different dimensions. A dimension threshold can be made when Accs and AUCs are keeping almost the same with dimension incrementally increasing. Thus, the variables that really help to recognize proteins with specific functions are selected from the encoded feature.

### Measure

Evaluation metrics are made to estimate the effectiveness of selected variables at the seventh step. The classification error rate is expressed as follows,

1 $$\begin{aligned} Err={{FN+FP} \over {TP+FN+TN+FP}}, \end{aligned}$$

where *TP*, *TN*, *FP* and *FN* represent the number of true positive, true negative, false positive and false negative, respectively. On the contrary, *Acc* is shown as follows,

2 $$\begin{aligned} Acc={{TN+TP} \over {TP+FN+TN+FP}}. \end{aligned}$$

Besides, we choose four widely used quantitative measurements. The confusion matrix illustrates *TP*, *TN*, *FP* and *FN* together. Besides, *Precision* and *Recall* are computed as follows,

3 $$\begin{aligned} Precision= & {} {{TP} \over {TP+FP}}, \end{aligned}$$

4 $$\begin{aligned} Recall= & {} {{TP} \over {TP+FN}}. \end{aligned}$$

In addition, $F1-measure$ is a harmonic average of *Precision* and *Recall*, which is expressed as

5 $$\begin{aligned} F1-measure = {{2*Precision*Recall} \over {Precision+Recall}}. \end{aligned}$$

Moreover, the ROC and AUC are also provided here as qualitative measurements.

## Data Availability

The public dataset analysed during the current study is available in reference [51], and can be downloaded from the website https://github.com/LoopGan/Effective-prediction-of-bacterial-type-IV-secreted-effectors.

## Abbreviations

Acc: Accuracy;
AUC: Area under curve;
DTC: Decision tree classifier;
GBM: Gradient boosting machine;
kNN: k-nearest-neighbor;
LDA: Linear discriminant analysis;
LR: Logistic regression;
MLP: Multi-layer perceptron;
NB: Naive bayesian;
PseAAC: Pseudo-amino acid composition;
PSI-BLAST: Position-specific iterated blast;
PSSM: Position-specific scoring matrix;
RF: Random forest;
ROC: Receiver operating characteristic;
SVM: Support vector machine;
T4SE: Type IV secreted effectors
